# Fostering clinical reasoning in physiotherapy: comparing the effects of concept map study and concept map completion after example study in novice and advanced learners

**DOI:** 10.1186/s12909-017-1076-z

**Published:** 2017-12-01

**Authors:** Katherine Montpetit-Tourangeau, Joseph-Omer Dyer, Anne Hudon, Monica Windsor, Bernard Charlin, Sílvia Mamede, Tamara van Gog

**Affiliations:** 1 School of Rehabilitation, Faculty of Medicine, Université de Montréal, P.O. Box 6128, Station Centre-Ville, Montreal, QC H3C 3J7 Canada; 2 Centre for Interdisciplinary Research in Rehabilitation of Greater Montreal (CRIR), Montreal, Canada; 3 Centre de pédagogie appliquée aux sciences de la santé (CPASS), Université de Montréal, Montreal, QC Canada; 4 Department of Neurology, Montreal General Hospital, Montreal, QC Canada; 5 Institute of Medical Education Research Rotterdam, Erasmus Medical Center, Rotterdam, The Netherlands; 6 Department of Psychology, Erasmus University Rotterdam, Rotterdam, The Netherlands; 7 Department of Education, Utrecht University, Utrecht, The Netherlands

**Keywords:** Clinical reasoning, Concept map, Cognitive load, Worked example, Near transfer, Far transfer, Physiotherapy

## Abstract

**Background:**

Health profession learners can foster clinical reasoning by studying worked examples presenting fully worked out solutions to a clinical problem. It is possible to improve the learning effect of these worked examples by combining them with other learning activities based on concept maps. This study investigated which combinaison of activities, worked examples study with concept map completion or worked examples study with concept map study, fosters more meaningful learning of intervention knowledge in physiotherapy students. Moreover, this study compared the learning effects of these learning activity combinations between novice and advanced learners.

**Methods:**

Sixty-one second-year physiotherapy students participated in the study which included a pre-test phase, a 130-min guided-learning phase and a four-week self-study phase. During the guided and self-study learning sessions, participants had to study three written worked examples presenting the clinical reasoning for selecting electrotherapeutic currents to treat patients with motor deficits. After each example, participants engaged in either concept map completion or concept map study depending on which learning condition they were randomly allocated to. Students participated in an immediate post-test at the end of the guided-learning phase and a delayed post-test at the end of the self-study phase. Post-tests assessed the understanding of principles governing the domain of knowledge to be learned (conceptual knowledge) and the ability to solve new problems that have similar (i.e., near transfer) or different (i.e., far transfer) solution rationales as problems previously studied in the examples.

**Results:**

Learners engaged in concept map completion outperformed those engaged in concept map study on near transfer (*p* = .010) and far transfer (*p* < .001) performance. There was a significant interaction effect of learners’ prior ability and learning condition on conceptual knowledge but not on near and far transfer performance.

**Conclusions:**

Worked examples study combined with concept map completion led to greater transfer performance than worked examples study combined with concept map study for both novice and advanced learners. Concept map completion might give learners better insight into what they have and have not yet learned, allowing them to focus on those aspects during subsequent example study.

**Electronic supplementary material:**

The online version of this article (10.1186/s12909-017-1076-z) contains supplementary material, which is available to authorized users.

## Background

Worked examples presenting the complete solution steps for solving clinical problems can be used for fostering clinical reasoning in health profession learners [1–3]. However, worked examples study alone is not optimal for fostering transfer of learning, that is a learner’s capacity to solve problems that are different from those studied in the examples [1]. It is unclear how worked example-based learning can be improved to foster greater transfer of learning, and in so doing, promote more meaningful learning. One strategy would be to combine worked examples with other learning activities to help learners deepen their understanding of the examples studied [4]. In this way, worked examples have been combined with other activities based on concept maps [5]. Concept maps can foster in-depth understanding by allowing learners to complete the missing parts of an incomplete concept map (concept map completion). Learners can alsostudy a fully worked out concept map that presents the links between the concepts relevant to understanding a specific field of knowledge (concept map study) [6, 7]. Providing learners who are studying worked examples with an additional activity involving concept maps might be a good way to improve the worked example learning effect. However, it is unclear which additional activity (i.e., concept map completion or concept map study) would foster more meaningful learning among learners engaged in worked example study. Moreover, some evidence suggests that worked example learning effects can be influenced by learners’ proficiency level in the skill being learned [8–11].Therefore, it is also unclear whether combinaisons of worked examples study with concept map activities will have similar learning effects on novice health profession learners who have little prior knowledge of the field studied, and on advanced learners who start gaining experience while actually coping with real clinical problems.

### Example-based learning

Example-based learning can foster clinical reasoning in health profession learners [1–3, 12]. This strategy can be put forward by using worked examples whereby learners acquire problem-solving skills by studying a written worked-out solution to a problem [13, 14]. For instance, learning from written worked examples presented in a digital learning environment was found to foster diagnostic knowledge in two studies with medical students [2, 3, 12]. Worked examples have shown to be more efficient, compared to conventional classroom teaching and learning activities based on problem-solving practice [14–16]. This so-called *worked example effect* is explained by cognitive load theory in terms of the cognitive demands imposed by worked example study vs. problem-solving practice [17]. Cognitive load theory stipulates that working memory has a limited capacity, and that activities which overload working memory can impede learning. Worked example study imposes less cognitive load and allows more room for fostering cognitive processes involved in effective learning compared to problem-solving practice [18]. In fact, worked examples are effective in allowing learners to acquire the very specific knowledge presented in the examples. However, learners engaged in worked examples study have difficulty using the knowledge acquired in the examples presented in new meaningful situations. This raises the question of how learning from worked examples can be improved to stimulate meaningful learning.

### Meaningful learning

In health science professions, meaningful learning implies that knowledge gained by learners makes sense in their future practice [19–21]. This can be refered to as transfer of learning, which is a learner’s capacity to apply the knowledge acquired to solve new problems. Transfer becomes increasingly difficult to achieve as the new situation varies from the learning situation. Near transfer, which occurs when the new situation is similar to the learning situation, is a more accessible educational goal than far transfer, which occurs when the two situations are dissimilar [22, 23]. Transfer of learning is an important educational goal, but notoriously difficult to achieve [24]. Notably, some evidence has shown that learning with worked examples is not sufficient for promoting transfer of learning. For instance, a study on history taking in physiotherapy students [1] revealed that examples based on the actual performance of an expert promoted better retention than didactical examples but had no significant effect on transfer performance. A simple approach to stimulating transfer in learners studying worked examples would be to provide additional learning activities that focus learners’ attention on the underlying principles and relationships of the problem solution presented in the examples [4]. For instance, concept maps are learning tools that can help learners deepen their understanding of a field of knowledge.

### Concept maps

Concept maps are instructional tools that promote individual thinking processes which, in turn, can foster meaningful learning [25–27]. Concept maps consist of concepts or ideas usually presented in cells or nodes, which are cross-linked by labelled lines or arrows that intend to show, in an explicit manner, the relationships between the concepts [28]. Concept mapping engages learners in active information processing in order to organize their knowledge within a concept map [28]. In health profession education, concept mapping was found to produce significant improvements in understanding and problem solving in learners when compared to traditional teaching methods [20, 29, 30]. Concept map *completion*, in which learners study partially worked-out concept maps and complete the missing parts, has been shown to foster meaningful learning in medical students [19].

One drawback of concept mapping and concept map completion is that they are likely to impose high cognitive load when learners have to deal with knowledge in complex fields such as reasoning about clinical cases. Studying model concept maps is another instructional approach that can potentially promote meaningful learning (focussing learners’ attention on the underlying problem structure) while imposing less load on working memory compared to concept mapping. There is a large body of evidence indicating that concept map study promotes information recall [31–36]. Moreover, expert concept maps as advanced organizers have been found to enhance a deeper understanding of medical knowledge among resident physicians [37].

### Combining worked examples with concept maps

It seems relevant to combine worked examples study with activities based on concept maps in fostering meaningful learning. The rationale behind this strategy is to improve the worked example effect by allowing learners to engage in activities based on concept maps by which they can study the in-depth structure of the examples. This raises the question of which combinaison, worked examples study with concept map completion or worked examples study with concept map study, is the the best for promoting meaningful learning. On one hand, concept map completion might be more effective because it allows learners to be more actively engaged in in-depth processing of the concepts relevant to the field of knowledge being studied compared to concept map study. On the other hand, concept map completion might be less effective because it imposes more cognitive load and is more likely to overload learner’s working memory compared to concept map study. A previous study by our team showed no difference between additional concept map completion and concept map study in fostering near transfer performance of problem-solving skills in novice physiotherapy students engaged in worked examples study [5].However, this previous study is limited by the fact that it included a single-learning session with only an immediate post-test, and it did not investigate far transfer. As learning tools, concept maps are often used in a repetitive manner over a certain period of time to potentiate their effects [27, 37]. It is possible that benefits of map completion are only demonstrated over time. Therefore, it would be relevant to compare, in learners engaged in worked examples study, the effects of additionally providing *repetitive* concept map completion and concept map study on meaningful learning.

### Novice versus advanced learners

An important issue for educators who are considering using worked examples is the fact that the effects are influenced by learners’ proficiency level. Worked examples are mainly effective in helping novice learners understand how to solve problems in structured domains, that is, fields of knowledge in which problem-solving tasks are rule based [18]. Research indeed shows that, in structured domains, as learners increase their proficiency in problem-solving, the worked example effect initially disappears and then reverses, resulting in impeded learning in most advanced learners [38, 39]. This so-called *expertise reversal effect* is explained by the fact that worked examples become redundant and thus impose more cognitive load on more advanced learners [11, 38]. More recent research, however, suggests that in less structured domains such as reasoning about legal cases [10] or clinical cases [40], both novice and advanced learners benefit from examples possibly because it takes longer to fully acquire less structured skills (i.e., an absence of the expertise reversal effect). In light of these studies, it seems relevant, in less structured domains (such as clinical reasoning in health professions), to compare the effects of learning activities based on worked examples between novice and advanced learners.

### Clinical reasoning in the physiotherapy domain

The specific learning domain addressed in the present study is clinical reasoning in the selection and use of therapeutic electrical currents in physiotherapy to treat patients with motor deficits (e.g., weakness, movement disorders). These currents can produce neuromuscular electrical stimulations that are used to treat patients with musculoskeletal, orthopaedic and neurological impairments [41–43]. The parameters of these currents (intensity, pulse frequency, pulse duration, electrode placement) must be adjusted according to patients’ physical impairments and the therapeutic objectives [44, 45]. It is possible to design process-oriented worked examples that present the clinical reasoning (i.e., decision-making process) underlying the selection of the optimal electrophysical agent to use in physiotherapy, and how to parametrize this agent [5].

In order to evaluate meaningful learning of clinical reasoning skills, one must consider the assessment of conceptual knowledge and problem-solving performance. Conceptual knowledge is the understanding of the principles governing a domain and the interrelations between units of knowledge [46]. Problem-solving ability can be assessed by near and far transfer performance. When assessing clinical reasoning, it is relevant to evaluate conceptual knowledge and problem-solving ability because these two types of knowledge can both influence decision-making skills [47, 48] but may not be related to one another [48–50].

#### Objectives

The first objective of this study was to investigate which learning condition, concept map study or concept map completion, would be more effective in promoting meaningful learning of physiotherapy intervention knowledge in learners engaged in worked examples study. It was expected that concept map completion would be most effective in fostering meaningful learning. This is because concept map completion allows learners to process the learning material more actively compared to concept map study. In this study, meaningful learning was assessed by learners’ capacity to solve problems that are different from those studied in the worked examples. This capacity was evaluated by the mean of near transfer and far transfer performance. Near transfer was evaluated by learners’ capacity to solve problems that are different, but require solutions that are similar to those they have studied in the examples [51]. Far transfer was evaluated by learners’ capacity to solve problems that require solutions that are different from those they have studied in the examples [51].

The second objective was to compare the learning effects of concept map study and concept map completion between novice and advanced learners engaged in worked examples study. It was expected that novice and advanced learners would similarly benefit from the learning activities because the learning task was ill structured. This hypothesis was based on previous studies showing that both novice and advanced learners similarly benefit from worked examples in ill-structured domains [10, 40]. In the present study, the task being learned, that is, selecting an optimal physiotherapy intervention for treating a patient with a physical impairment, is an ill-structured task because the aim to achieve is not always well defined and multiple paths might lead to an acceptable solution [5, 52]. In contrast, in well-structured domains such as mathematics or physics, problem-solving is mainly rule-based [52]. In these well structured domains, novice learners are expected to benefit more from worked examples than advanced learners [5, 52].

The third objective was to compare the cognitive load imposed by the learning activity between concept map study and concept map completion in learners engaged in worked examples study. It was expected that the concept map study condition would be cognitively less demanding than concept map completion. This is because learners engaged in concept map completion had to perform tasks such as generating and organizing concepts that are cognitively more demanding than the cognitive tasks associated with concept map study, that is, studying concepts and their relationships. In this study, the cognitive load imposed by the learning activity was estimated by the perception of the mental effort invested by learners during the activity [53].

The fourth objective was to compare the cognitive load imposed by the post-tests assessing learning outcomes between the concept map study and the concept map study completion conditions. It was expected that the cognitive load while performing these tests would be less important in the concept map completion condition than concept map study. This is because learners engaged in concept map completion might have achieved more in-depth learning and acquired more cognitive schemas of the problem-solving task than those engaged in concept map study. These cognitive schemas might facilitate test performance in learners engaged in concept map completion compared to those engaged in concept map study. As for the cognitive load imposed by learning activities, the cognitive load imposed by post-tests was estimated with the subjective evaluation of the mental effort invested by learners during these tasks [53].

## Methods

### Participants

All second-year physiotherapy students (*n* = 93) from Université de Montréal (Canada) were invited to participate in the study. The activities of the present study were part of the students’ academic curriculum and counted toward a course credit. Students were considered eligible based on experience with previous cohorts in this program, demonstrating that, at this stage of their training, second-year students have heterogeneous proficiency levels in selecting electrotherapy interventions to treat patients with motor deficits. In fact, experience with previous cohorts of this program shows that half of second-year physiotherapy students have little knowledge about this skill and that the other half has acceptable performance when performing that skill. The reason for this variability is that some students have previous clinical experience as physiotherapist assistants, have received training in other fields related to physiotherapy (e.g., kinesiology) before being enrolled in the physiotherapy program, or have done previous rotations during which they had to use electrotherapeutic currents to treat patients with movement disorders.

Before the study took place, all participants attended a three-hour instructional course on concept mapping using the free IHMC Cmap software tool available online (http://cmap.ihmc.us/) as part of their training during their first year in the program. No participant had ever participated in prior educational research involving worked examples or concept mapping. Of the ninety-three eligible students, sixty-one (44 women; mean age ± SD: 22.6 ± 3.0 years) gave their informed consent to participate. Thirty-two did not gave their consent to participate in the study, and therefore their data was not assessed. The ethics committee of Université de Montréal’s Faculty of Medicine approved the study.

### Study design

**Fig. 1:**
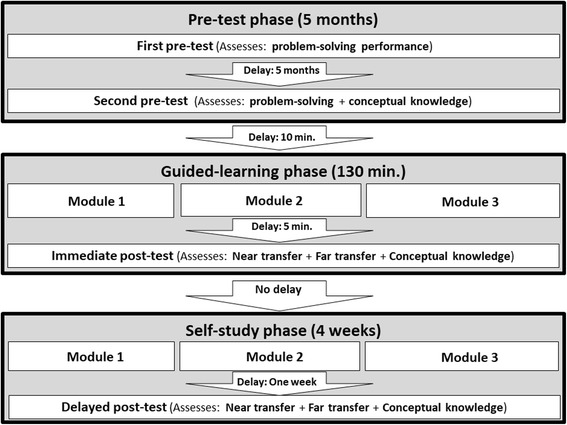
Flowchart of study activities

This study included three phases. Students participated in 1) a five-month pre-test phase, 2) a 130-min guided-learning phase, and 3) a four-week self-study phase (Figure 1). The pre-test phase consisted in an initial fifty-minute pre-test, and five months later, a second fifty-minute pre-test. Participants individually completed all pre-tests in the digital learning environment available at Université de Montréal. The first pre-test assessed students’ problem-solving skills in selecting appropriate electrotherapeutic currents to treat patients with motor deficits. The first pre-test aimed at categorizing students according to their proficiency in this problem-solving skill. Students in the novice or advanced learners group had undergone an initial categorization according to the results of the first pre-test. Details pertaining to this categorization are explained in the next section. The second pre-test also assessed students’ problem-solving skills and was performed to test categorization of participants based on the results of the first pre-test. The second pre-test was performed just before students participated in the learning activities of the guided and self-directed learning phases. To compare problem-solving ability between novice and advanced learners during the pre-test phase, a 2 × 2 factorial design was used with “Learner’s Prior Ability” (novice vs. advanced learners) as a between-subjects factor and “Time Moment” (first vs. second pre-tests) as a within-subjects factor.

**Fig. 2:**
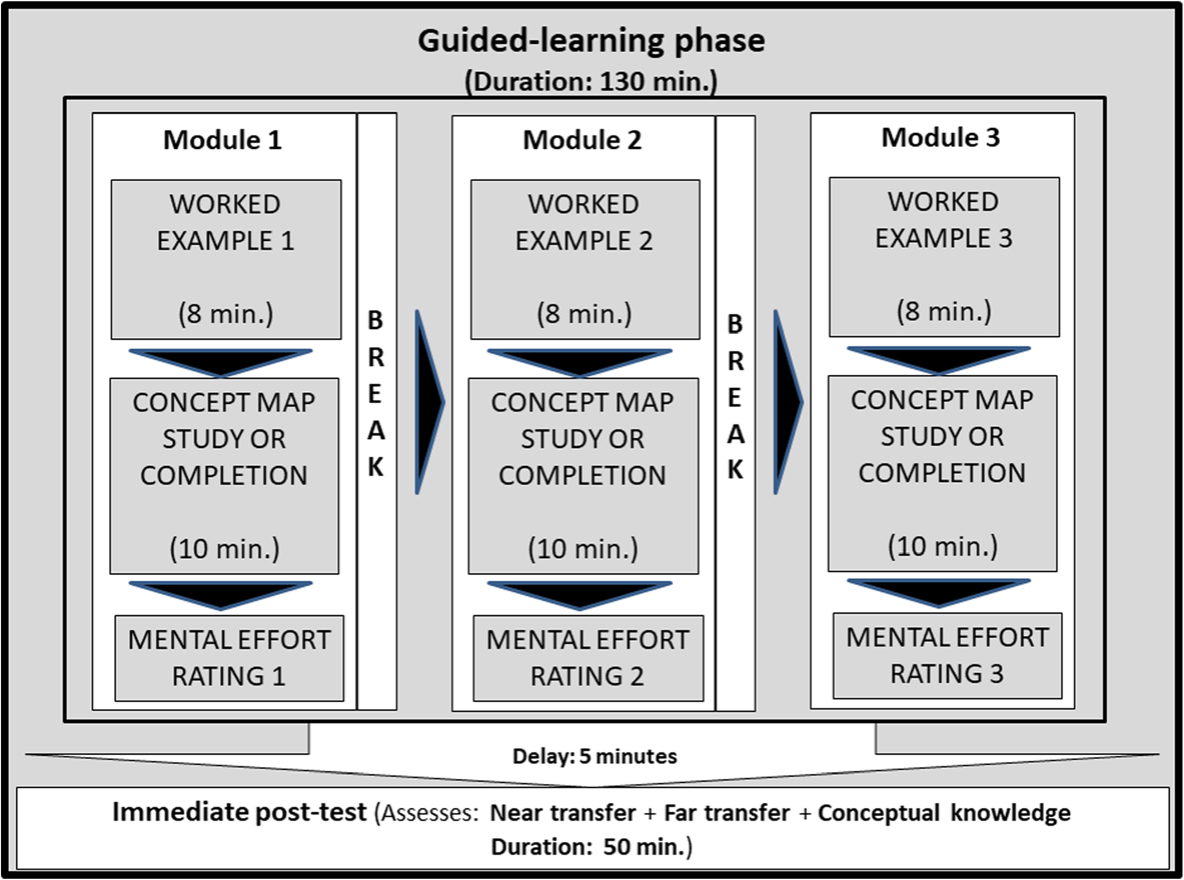
Flowchart of guided-learning phase activities

After the pre-test phase, students categorized as novice or advanced learners were then randomly assigned to either the concept map completion condition or the concept map study condition. Then, participants participated in a 130-min guided-learning phase. This phase included an 80-min online learning session with worked examples and activities based on concept maps (i.e., concept map completion or study), followed by a 50-min immediate post-test (Fig. 2). Then, students participated in a four-week self-study phase. During this phase, students had unlimited online access for three weeks to the learning activities performed during the guided-learning phase. One week later, students participated in a 50-min delayed post-test. Participants individually completed all the post-tests and learning activities in the digital learning environment. To assess learning outcomes during the guided-learning phase and the self-study phase, a 2 × 2 × 2 factorial design was used with “Learning Condition” (concept map completion vs. concept map study) and “Learner’s Prior Ability” (novice vs. advanced learners) as between-subject factors and “Time Moments” (immediate vs. delayed post-tests) as a within-subjects factor.

### Pre-test phase

#### Model for categorizing participants’ proficiency level

The Dreyfus skill acquisition model was used to categorize participants according to their proficiency level for the skill to bebeing learned before they participated in the learning activities [54, 55]. According to this model, learners can be categorized as novice, advanced beginner, competent, proficient or expert. Novice learners have little to no knowledge of the task being learned, whereas advanced beginners start coping with real situations and can show marginally acceptable performance. Learners are considered competent when they consistently show an acceptable level of performance in the skill being learned. As competent learners gain experience, they show increasing levels of performance and eventually become proficient learners, even experts. Based on the Dreyfus model and on experience with previous cohorts of students, it was expected that each participant in this cohort would be categorized as either a novice, or as a learner with a higher skill level than a novice (i.e., advanced learner which could potentially include the advanced beginner, competent, proficient and expert levels of Dreyfus model). Based from experience with previous cohorts, it was expected that novice learners would have a mean performance below 60%, whereas advanced learners would have a mean performance above 60% of the maximum in the first pre-test. Futhermore, based on previous experience, it was expected that advanced learners would have more than one month of clinical experience in the skill being learned in this study. Therefore, it was decided a priori that participants would undergo an initial categorization based on the results of the first pre-test. They would be categorized as novice learners if they were unable to achieve an acceptable level of performance during the first pre-test (i.e., mean performance below 60% across pre-tests). Conversely, participants would be categorized as advanced learners, if they showed at least marginally acceptable performance (i.e., mean performance of 60% or more in the first pre-test), and if they have at least one month of previous relevant clinical experience in performing the task [56]. This categorization would be confirmed if advanced learners continued to outperform novices, and if novices did not gain more than one month of clinical experience in performing the task at the end of the pre-test phase. The amount of practice required for novice learners to achieve the level of advanced beginners depends, among other things, on the complexity of the skill being learned. Therefore, this amount of practice varies across the skills studied. In this way, studies in health profession education have reported learners who were considered advanced beginners after practicing the skill for less than 25 h to up to one year [57–59]. For the purpose of the present study, it is relevant to categorize participants between novice learners and those with a higher skill levels(i.e., advanced learners). This is because the transition from novice to advanced beginners is a key step when considering the development of problem-solving skills in ill-structured clinical domains [60]. In fact, this transition is a tipping point from which learners starts to be able to use non-analytic reasoning processes that allow them to efficiently perform ill-structured problem-solving tasks such as the one used in the present study. It was possible to categorize students from the same academic level, that is, second-year physiotherapy students, into novice and advanced learners in the skill being learned because such clinical reasoning skill depends more on clinical experience rather than on students’ academic level [55, 60, 61]. This is in accordance with previous studies in which students of the same academic level showed different levels of expertise when performing a specific skill [61–63].

#### First pre-test

The first pre-test consisted of problem-solving tasks in the form of four clinical cases presenting a patient with a physical impairment causing a movement disorder. After reading each case, participants had to answer the following short-answer questions: “What is the most appropriate electrotherapy intervention in this case?” (1 point) and “What are the optimal adjustment parameters for the electrotherapy intervention selected?” (2 points) (see Appendix A). The maximum score on this test was twelve. Reliability of the prior ability test was sufficient for group comparison (Internal consistency with twelve questions: Cronbach’s α = 0.89). The score obtained was converted to a percentage. Short-answer questions on diagnoses and therapy recommendations based on a patient case study, such as those used in the tests of the present study, can assess clinical reasoning [64–67]. The first pre-test also included other suitable questions on other topics (i.e., use of electrophysical agents in physiotherapy other than electrotherapeutic currents) randomly presented. Moreover, the first pre-test also included three questions about students’ previous training related to physiotherapy and the amount of clinical experience with electrophysical agents. These questions read as follows: “Did you have any previous training related to physiotherapy before your admission to the current physiotherapy program (Yes/No)?”; “What was your previous training related to physiotherapy/rehabilitation before the current physiotherapy program (eg., BSc in Kinesiology, physiotherapist assistant)?”; “Provide the amount of clinical experience (in months) you have (including rotations, work with sports teams, volunteer work) in selecting electrophysical agents to treat patients with movement disorders.” Assessement of the results of this test and the short questionnaire confirmed that each second-year physiotherapy student could be categorized as either a novice or advanced learner. Twenty-nine students were assigned to the advanced learners group (22 women; mean age ± SD: 23.6 ± 3.9 years) because they met both of the following conditions: they showed acceptable performance in the skill being learned by scoring 60% or more on the first pre-test, and theystated having had more than one month of previous experience in using electrophysical agents to treat patients with movement disorders. The rest of the participants, that is, thirty-two participants, were assigned to the novice learners group because they scored less than 60% (22 women; 21.7 ± 1.6 years). Age was significantly higher in participants assigned to the advanced learners group than in those assigned to the novice learners group (Student’s t (59) = 2.482; *p* = .016, *two-tailed*).

#### Second pre-test

The second pre-test assessed problem-solving performance and conceptual knowledge, and also included the same three open questions about students’ previous training and clinical experience as used in the first pre-test. Problem-solving performance was measured with four tasks, consisting of a short case study scenario and the following questions for students to answer: 1) “What is the most appropriate electrotherapy intervention in this case?” (1 point); 2) “What are the optimal adjustment parameters for the electrotherapy intervention selected?” (2 points); and 3) “What key characteristics of the case justify the intervention selected?” (2 points) (see Appendix D). These cases were different from those used in the first pre-test. The maximum score was 20 points (Internal consistency with twelve questions: Cronbach’s α = 0.87). Conceptual knowledge was evaluated with five multiple-choice questions with one best answer (2 points) or an alternative answer accepted (1 point) (see Appendix C). The maximum score was ten points (Internal consistency with five questions: Cronbach’s α = 0.73). Conceptual knowledge can be assessed using multiple choice questions [68, 69] when these questions assess learners’ understanding of principles governing a field of interest and the interrelations between units of knowledge [46].

#### Comparison of prior ability between novice and advanced learners

In order to test the categorization of novice and advanced learners after the results of the first pre-test, analyses were performed to compare problem-solving performance between the two groups across the two pre-tests. Analyses showed that there was a significant main effect of learners’ prior ability (large effect size) on problem-solving performance during the pre-test phase (Table 1). Follow-up t-tests on problem-solving performance averaged across the two pre-tests indicated that mean performance in the advanced learners group (Mean ± SD: 70.7 ± 14.8%) was higher (t (60) = 8.838, p < .001, two-tailed, Cohen’s d = 2.281: large effect size) than in the novice learners group (44.7 ± 11.2%). Advanced learners showed higher problem-solving performance than novice learners on the first pre-test (t (59) = 12.665, p < .001, two-tailed, Cohen’s d = 3.236: large effect size) and on the second pre-test (t (59) = 18.713, p < .001, two-tailed, Cohen’s d = 4.872: large effect size) (Table 1). There was a significant main test moment effect (medium effect size) on problem-solving performance, with a tendency of higher mean performance across groups on the first pre-test (59.0 ± 20.4%) than on the second pre-test (55.9 ± 16.4%). There was also a significant learners’ prior ability x test moment interaction effect (large effect size) on problem-solving performance. Follow-up t-tests on problem-solving performance showed that problem-solving performance in novice learners was lower on the first pre-test than on the second pre-test (t(31) = 2.552, *p* = .016), whereas in advanced learners performance was higher on the first pre-test than on the second pre-test (t (28) = 6.025, p < .001). To assess whether there was a difference in conceptual knowledge performance before the learning activities according to learners’ prior ability, a t-test was performed to compare conceptual knowledge on the second pre-test between novice and advanced learners. This test revealed a non-significant difference between novice and advanced learners in conceptual knowledge on the second pre-test (t (59) = .774; *p* = .442). In order to assess whether there could be changes in the categorization of participants based on their clinical experience in the skill being learned during the pre-test phase, their answers in the second pre-test to three questions about students’ previous training related to physiotherapy and the amount of clinical experience in using electrophysical agents were assessed. This assessement showed that participants’ answers in the first pre-test were consistnt with their answers on the second pre-test and that no participant gained significant clinical experience during the pre-test phase. This analysis confirmed that students can be categorized in two prior ability groups (i.e., novice and advanced learners) and that no students changed their prior ability level category during the pre-test phase. Within each prior ability level, students were randomly assigned to either the concept map completion condition (novice learners: *N* = 16; advanced learners: *N* = 15) or the concept map study condition (novice learners: N = 16; advanced learners: *N* = 14).

**Table 1:** Mean problem-solving performance (SD) across the first and second pre-test of the pre-test phaseexpressed as a percentage of the maximum score, and 2 × 2 ANOVA results for the effects of learners’ prior ability, time moment and factor interaction

Learning outcome	Advanced learners			Novice learners			2 × 2 ANOVA			
								Main effects		Interaction effect
	First pre-test	Second Pre-test	Mean	First pre-test	Second Pre-test	Mean	Statistical value	Prior ability	Test moment	Prior ability x Test moment
Problem-solving performance	77.1 (11.9)	65.8 (14.9)	70.7 (14.8)	42.7 (9.3)	47.1 (12.3)	44.7 (11.2)	F_(1,57)_	86.840	7.306	38.057
							MSE	21,492.292	361.266	1881.896
							P	**< .001**	**= .009**	**< .001**
							η_p_^2^	.595	.110	.392

MSE represents mean-square error value; η_p_^2^ represents effect size (partial eta square) calculated for all comparisons. Statistically significant *p* values (*p* < .05) are presented in bold

### Guided-learning phase

After the second pre-test in the pre-test phase, students had a ten-minute break and then participated in a 130-min. Guided-learning phase. All activities in this phase were monitored and students performed them individually in the digital learning environment at the university. Figure 2 presents the timeline of the activities performed during the guided-learning phase. Firstly, participants had to participate in three learning modules presented in random order. Each module consisted of a worked example and an additional integrative learning activity (concept map completion or concept map study depending on the assigned condition). Each worked example was presented for eight minutes with the following instructions: “You have 8 minutes to carefully study the following example.” The development procedure of worked examples is described in the Materials section. After each worked example, students had 10 min to participate in the integrative activity, depending on their randomly assigned condition. This activity involved 1) completing a concept map that represented the principles of intervention selection from a sheet on which key concepts were already presented (Concept map completion condition); or 2) studying a model concept map presenting the principles of intervention selection (concept map study condition). In the concept map completion condition, participants constructed a concept map throughout the guided learning phase. Similarly, for the concept map study condition, participants studied a model concept map throughout the guided learning phase. The concept map development procedure is described in the Materials section. Directly after each activity (i.e., example study, concept map study or concept map completion) participants had to rate the mental effort they invested while working through the activity. The mental effort rating scale is described in the Materials section. Students took a 5-min break after completing each module. Then they had fifty minutes to complete the immediate monitored post-test, after which they rated the mental effort invested in the test. The details of the immediate post-test are described in the Materials section. The duration of each activity was pilot-tested with students from a previous cohort who did not participate in the present study.

### Self-study phase

After completing the immediate post-test in the guided-learning phase, students were instructed to participate in the self-study phase. During the four-week self-study phase, students were allowed to participate in a three-week, self-study session during which they could access the modules online from anywhere, at any time and for as long as they wanted. They were instructed not to work together or help each other while performing the self-study phase and not to discuss the material or activities they were involved in during the guided and self-study phases. Students in the concept map completion group had access to the concept map they worked on during the guided learning session. They were instructed to continue improving their concept map. Students in the concept map study group had access to the model concept map they studied during the guided learning session. They were instructed to continue studying the model concept map. The digital learning environment recorded and compiled the duration of each log-on session for each participant. All participants were instructed to spend at least seventy minutes on completing the learning activities of the self-study phase, which is, at the very least, the amount of time they spent on the modules (examples + concept map) in the guided learning session. One week after the self-study session, students took part in a monitored delayed post-test at the university and had a maximum of ninety minutes to complete this test. The details of the delayed post-test are described in the Materials section.

### Materials

#### Worked examples

Three worked examples were used in the learning session for this study. The development procedure for these examples was described in another study [5]. The worked examples were process-oriented and presented clinical cases of patients admitted to physiotherapy for a physical impairment resulting in motor deficits (e.g., weakness, lack of coordination, motor control deficits). The worked examples were designed according to May and Newman’s sequence of actions for solving physiotherapy problems and the SOAP note model [70, 71]. This sequence of actions includes: problem recognition, problem definition, problem analysis, data management, solution development and solution implementation [70, 72]. The worked examples integrated these actions and were presented in the form of a SOAP note. The SOAP note model, which includes reporting Subjective and Objective information, Assessment of the problem and Plan for treatment, is widely used in various health professions [71, 73].

Each written worked example (see Appendix B) described a) the clinical problem of a patient in consultation with a physiotherapist; b) the physiotherapist’s detailed clinical reasoning, raising several hypotheses about potential electrotherapeutic currents and explaining why they were or were not suitable for the case; c) the electrotherapeutic current selected by the physiotherapist; d) the application parameters of the electrotherapeutic current chosen (e.g., electrode placement, intensity, stimulation frequency, duration of treatment); and e) the key elements of the clinical case and context justifying the intervention selected [5].

#### Concept maps

For the concept map completion condition, one of the co-authors (JOD) created an incomplete concept map using the free IHMC Cmap software tool available online (http://cmap.ihmc.us/). This incomplete map only presented concepts in boxes with rounded edges without presenting the links explaining the relationships between the concepts. The presented concepts were the main concepts of the decision-making processes used to select the type of electrotherapeutic current to treat a motor deficit. These concepts were similar to those provided in the worked examples and were chosen based on their relevance in answering the specific question presented at the top of the map [5]. The main question for the incomplete concept map reads as follows: “How do you choose an appropriate electrotherapeutic current for a motor deficit?” (see Additional file 1).

As for the concept map study condition, the second author created a model concept map using the same software as the incomplete map. This map presented a visual diagram of the decision-making process used to select the electrotherapeutic current to treat problems associated with motor deficits [5]. Hierarchical concept maps present concepts in a ranking order, with most general and inclusive concepts at the top of the map and more specific concepts arranged below. The model concept map incorporated the information provided by the worked examples. It was developed to answer a specific question, which was presented at the top of the map. The main question reads as follows: “How do you choose an appropriate electrotherapeutic current for a motor deficit?” (see Additional file 2).

#### Immediate post-test

The immediate post-test evaluated conceptual knowledge, near transfer performance and far transfer performance. Conceptual knowledge on the immediate post-test was evaluated with five multiple-choice questions that shared the same format but were different from those of the second pre-test. The maximum score was ten points (Internal consistency with five questions: Cronbach’s α = 0.72). Near transfer was evaluated with four cases that had the same format and required the same treatment as the four problem-solving tasks in the second pre-test and the worked examples in the learning session, but had different cover stories (i.e., story of patient’s main complaint). The maximum score was 20 points (Internal consistency with twelve questions Cronbach’s α = 0.80). In this study, near transfer refers to learners’ capacity to solve new problems that require problem-solving strategies that are similar to the strategies they have studied. Near transfer performance can be assessed by requiring learners to solve problems that show different superficial characteristics (i.e., information that is not required to find problem-solution strategies), but similar deep structure (i.e., solution procedure) compared to the problems they have learned [13, 14, 74]. Far transfer in the immediate post-test was evaluated with three tasks that had the same format as the near transfer tasks (see Appendix E). However, the cover stories were different and required different treatments from those presented in the worked examples. The maximum score was 15 points (Internal consistency with nine questions: Cronbach’s α = 0.71). In this study, far transfer refers to learners’ capacity to solve new problems that require problem-solving strategies that are different from the strategies they have studied. Far transfer performance can be assessed by requiring learners to solve problems that show different superficial characteristics and deep structure compared to the problems they have learned [13, 14, 75].

#### Delayed post-test

The delayed post-test evaluated conceptual knowledge, near transfer performance and far transfer performance. Conceptual knowledge on the delayed post-test was evaluated with ten multiple-choice questions that shared the same format but were different from those of the immediate post-test. The maximum score was 20 points (Internal consistency with ten questions: Cronbach’s α = 0.64). Near transfer was evaluated with six tasks that were comparable to those on the immediate post-test (and worked examples) but again had different cover stories. The maximum score was 30 points (Internal consistency with eighteen questions: Cronbach’s α = 0.77). Far transfer was evaluated with six tasks that were comparable to those on the immediate post-test but with different cover stories. The maximum score was 30 points (Internal consistency with eighteen questions: Cronbach’s α = 0.71). All test tasks showed sufficient internal consistency reliability for group comparison (Cronbach’s α ≥ 0.60). All scores obtained in these tests were converted to percentages to allow for comparison.

#### Mental effort rating scale

The mental effort invested in the learning activities and the post-tests was evaluated after each task with a nine-point subjective rating scale ranging from 1, which represented very, very low mental effort to 9, which represented very, very high mental effort. This scale, developed by Paas (1992), is widely used in educational research [1, 76]. Mental effort ratings provide an indication of the cognitive load experienced by the learner while performing a task [77, 78].

### Learning environment

The pre-tests, post-tests, worked examples, concept map tools, concept maps and mental effort rating scales were presented to the participants within the Université de Montréal’s digital learning environment. This environment was designed based on the e-learning Moodle platform (version 2.5). All participants were familiar with this learning environment given that they had been using it regularly for over a year in other courses. Answers to tests and mental effort rating scales were entered into the learning environment and analyzed a posteriori.

### Data analysis

#### Tests scoring procedure

Problem-solving performance on the pre- and post-tests was scored using the same procedure. For each question, a pre-determined checklist of acceptable responses and a scoring grid associated with these responses were used. The checklist was drafted by the physiotherapist who conducted the interviews with the expert physiotherapists, and was further revised by an experienced physiotherapy instructor. In order to determine the reliability of the scoring procedure, two independent raters unaware of the experimental procedure scored approximately 10% of the data (i.e., 7 participants). The intra-class correlation coefficients (ICC) for the two independent evaluators were 0.94 for the pre-test, 0.93 for the immediate post-test and 0.95 for the delayed post-test. Due to these high correlations, the rest of the scoring was done by only one of the raters whose scores were used in the analysis. All test results were presented as a percentage of the maximum score to allow comparison between the tests.

#### Assessment of learning outcomes

To check for differences in learning outcomes according to learning condition (cf. first objective of the study) and learners’ prior ability (cf. second objective of the study) as between-subjects factors, and test moment (immediate vs. delayed post-tests) as a within-subjects factor, 2 × 2 × 2 mixed ANOVAs were conducted on conceptual knowledge, near transfer and far transfer performance.

#### Assessment of mental effort invested

To assess differences in mental effort investment during learning as a function of the learning condition (cf. third objective of the study) and learners’ prior ability factors, a 2 × 2 ANOVA was performed on the mental effort invested during the guided-learning session. To check for differences in the mental effort associated with post-tests according to the learning condition (cf. fourth objective), learners’ prior ability and test moments, a 2 × 2 × 2 mixed ANOVA was performed on the effort invested across post-tests.

#### Assessment of time on task during the self-study phase

To assess differences in time on task during the self-study phase as a function of the learning condition and learners’ prior ability factors, a 2 × 2 ANOVA was performed on the log-on time during the self-study session.

Effect sizes were measured using partial η^2^ with .01, .06 and > .14 considered as weak, medium and large effect sizes, respectively [79]. *P* values ≤ .05 were considered significant. Student t-tests were performed as follow-up tests when ANOVAs showed significant effects. Effect sizes of significant differences on t-tests were measured using Cohen’s d with .2, .5 and .8 considered as weak, medium and large effect sizes, respectively [79]. Statistical analyses were performed using the Statistical Package for Social Science (SPSS) software (version 19 for Windows).

## Results

### Effects of the learning condition: Concept map completion vs. concept map study

Analyses were performed to compare the learning effects of concept map study and concept map completion for fostering far transfer, near transfer and conceptual knowledge (cf. first objective of the study). There was a significant main effect (large effect size) of the learning condition on far transfer performance across both post-tests, with concept map completion outperforming the concept map study condition (Table 2). There was no learning condition x test moment interaction effect on far transfer performance across post-tests. Analyses of near transfer performance across both post-tests showed a significant main effect of the learning condition (medium effect size), with concept map completion outperforming the concept map study condition (Table 2). There was no learning condition x test moment interaction effect on near transfer across post-tests. Analyses of conceptual knowledge across both post-tests showed no significant main effect of the learning condition (Table 2). However, there was a significant learning condition x test moment interaction effect (medium effect) on conceptual knowledge (Table 2). Follow-up t-tests indicated that on the immediate post-test, conceptual knowledge (60.7 ± 17.2%) was higher in the concept map study condition than in the concept map completion condition (50.6 ± 18.4%, *t* (59) = 2.194, *p* = .032, two-tailed, Cohen’s d = .567, medium effect size). However on the delayed post-test (i.e., the post-test, after the self-directed study session), conceptual knowledge was not significantly different between the two conditions (concept map completion: 87.4 ± 10.0%; concept map study: 86.0 ± 9.7%, *t*(59) = .563, *p* = .575, two-tailed) (Table 2).

**Table 2:** Mean conceptual knowledge, near transfer and far transfer performance (SD) across post-tests expressed as a percentage of the maximum score, and 2 × 2 × 2 ANOVAs results for the effects of learners’ prior ability, learning condition factors, time moment and factor interactions

	Learning condition						Learners’ prior ability						2 × 2 × 2 ANOVA						
	Concept map completion			Concept map study			Advanced learners			Novice learners				Main effects			Interaction effects		
	Post-Test			Post-test			Post-test			Post-test							Learning condition	Learning condition	Prior ability
Learning outcome	1	2	Mean	1	2	Mean	1	2	Mean	1	2	Mean	Statistical value	Learning condition	Prior ability	Test moment	X Prior ability	X Test moment	X Test moment
Far transfer	48.7 (16.1)	69.0 (10.9)	58.9 (17.1)	35.3 (18.0)	59.3 (18.5)	47.6 (21.7)	47.9 (18.7)	65.1 (15.4)	56.1 (18.9)	36.9 (16.4)	63.4 (16.3)	50.3 (21.3)	F_(1.57)_	13.341	3.991	73.715	.368	.604	3.128
													MSE	3899.674	1166.544	14,660.814	107.652	120.185	662.157
													P	**<.001**	.051	**< .001**	.546	.440	.082
													η_p_^2^=	.190	.065	.564	.006	.010	.052
Near transfer	67.1 (16.6)	89.3 (6.0)	78.3 (16.7)	58.1 (20.6)	81.5 (14.1)	70.1 (21.1)	69.6 (19.4)	86.6 (10.8)	78.0 (17.6)	56.4 (16.6)	84.4 (11.9)	70.4 (20.4)	F_(1.57)_	7.028	6.055	108.298	.120	.070	6.367
													MSE	2025.999	1745.678	15,470.321	34.722	9.970	909.495
													P	**.010**	**.017**	**< .001**	.730	.793	**.014**
													η_p_^2^=	.110	.096	.665	.002	.001	.100
Conceptual knowledge	50.6 (18.4)	87.4 (10.0)	69.0 (23.7)	60.7 (17.2)	86.0 (9.7)	73.6 (18.8)	57.9 (18.6)	85.5 (10.9)	71.9 (20.3)	53.4 (18.2)	87.8 (8.7)	70.6 (22.6)	F_(1.57)_	2.839	.218	158.467	3.666	5.637	2.048
													MSE	637.934	48.878	28,969.414	823.907	1030.472	377.392
													P	.097	.643	**< .001**	**.**061	**.021**	.158
													η_p_^2^=	.047	.004	.735	.060	.090	.035

1 represents Immediate post-test; 2 represents delayed post-test; Mean represents mean results across immediate and delayed post-tests. MSE represents mean-square error value; η_p_^2^ represents effect size (partial eta square) calculated for all comparisons. Statistically significant p values (*p* < .05) are presented in bold

### Effects of learners’ prior ability: Novice vs. advanced learners

Analyses were performed to compare the learning effects between novice and advanced learners on far transfer, near transfer and conceptual knowledge (cf. second objective of the study). Analyses of far transfer performance across both post-tests showed no main effect of learners’ prior ability and no significant interaction effect (Table 2). Analyses of near transfer performance across both post-tests showed a significant main effect of learners’ prior ability (medium effect size), with advanced learners outperforming novice learners (Table 2). There was no interaction effect of learners’ prior ability x learning condition on near transfer performance across post-tests. However, analyses showed a significant learners’ prior ability x test moment interaction effect (large effect size) on near transfer performance (Table 2). Follow-up t-tests indicated that advanced learners (69.6 ± 19.4%) outperformed (*t*(59) = −2.87, *p* = .006, two-tailed) novice learners (56.4 ± 16.6%) on the immediate post-test but no longer on the delayed post-test (after the self-directed study phase; advanced: 86.6 ± 10.8%, novice: 84.4 ± 11.9%; *t*(59) = .748, *p* = .457, two-tailed). Analyses of conceptual knowledge across both post-tests showed no significant effect of learners’ prior ability. However, there was a significant learners’ prior ability x learning condition interaction (medium effect size) (Table 2). Follow-up t-tests on conceptual knowledge averaged across both tests, indicated that for advanced learners, concept map study (76.8 ± 7.8%) was more effective (*t*(27) = 2.532, *p* = .017, two-tailed, Cohen’s d = .949, large effect) for conceptual knowledge acquisition than concept map completion (67.0 ± 12.4%). However, for novice learners, there was no effect of the learning condition on conceptual knowledge (concept map completion: 70.9 ± 10.2%; concept map study: 70.3 ± 11.3%; *t*(30) = .164, *p* = .871, two-tailed).

### Mental effort invested in the guided-learning activity

Analyses were performed to compare the mental effort invested by learners in the guided-learning activity between concept map study and concept map completion (cf. third objective of the study). There was a main effect of the learning condition (large effect size) on the mental effort invested by learners in the guided learning activity, with higher mental effort invested in concept map completion than in concept map study (Table 3). There was no significant main effect of learners’ prior ability and no prior ability x learning condition interaction effect on the mental effort invested during the guided learning activity.

**Table 3:** Mean mental effort invested (SD) in the guided learning activity, and 2 × 2 ANOVA results for the effects of learners’ prior ability, learning condition and factor interaction

	Learning condition		Learners’ prior ability		2 × 2 ANOVA			
						Main effects		Interaction effect
	Concept map completion	Concept map study	Advanced learners	Novice learners	Statistical value	Learning condition	Prior ability	Learning condition x Prior ability
Mental effort invested (max =9)	7.1 (0.9)	6.3 (1.1)	6.6 (1.0)	6.9 (1.2)	F_(1.57)_	9.048	.944	.475
					MSE	9.796	1.076	.515
					P	**.004**	.323	.493
					η_p_^2^	.137	.017	.008

MSE represents mean-square error value; η_p_^2^ represents effect size (partial eta square) calculated for all comparisons. Statistically significant p values (*p* < .05) are presented in bold

### Mental effort invested in the post-tests

Analyses were performed to compare the mental effort invested by learners across post-tests between concept map study and concept map completion (cf. fourth objective). There was no significant main effect of the learning condition or of learners’ prior ability on the mental effort invested by learners across post-tests (Table 4). However, there was a main learners’ prior ability x test moment interaction effect (medium effect size) on the mental effort invested across post-tests. Follow-up t-tests showed that novice learners invested more mental effort (7.2 ± .9) than advanced learners (6.4 ± 1.1, t(59) = 3.326, *p* = .003, Cohen’s d = .782, large effect size) on the immediate post-test, whereas this difference was not observed with the delayed post-test (novice: 6.3 ± 1.9; advanced: 6.6 ± 1.4, *t*(59) < 1, *p* = .477). There were no other significant interaction effects on the mental effort invested in the post-tests (Table 4).

**Table 4:** Mean mental effort invested (SD) during the immediate and delayed post-tests, and 2 × 2 × 2 ANOVA results for the effects of learners’ prior ability, learning condition factors, time moment and factor interactions

	Learning condition						Learners’ prior ability						2 × 2 × 2 ANOVA						
	Concept map completion			Concept map study			Advanced learners			Novice learners				Main effects			Interaction effects		
	Post-test			Post-test			Post-test			Post-test				Learning condition	Prior ability	Test moment	Learning condition	Learning condition	Prior ability
	1	2	Mean	1	2	Mean	1	2	Mean	1	2	Mean	Statistical value				X Prior ability	X Test moment	X Test moment
Mental effort invested (max. =9)	6.8 (1.0)	6.2 (1.5)	6.5 (1.3)	6.9 (1.1)	6.7 (1.8)	6.8 (1.5)	6.4 (1.1)	6.6 (1.4)	6.5 (1.3)	7.2 (0.9)	6.3 (1.9)	6.8 (1.5)	F_(1.57)_	1.131	.657	2.375	.862	1.092	6.308
													MSE	2.742	1.593	3.367	2.091	1.548	8.941
													P	.292	.421	.129	.357	.300	**.015**
													η_p_^2^=	.019	.011	.040	.015	.019	.100

1 represents Immediate post-test; 2 represents delayed post-test; Mean represents mean results for immediate and delayed post-tests. MSE represents mean-square error value; η_p_^2^ represents effect size (partial eta square) calculated for all comparisons. Statistically significant p values (*p* < .05) are presented in bold

### Log time during the self-study session

We also checked for possible differences in time on task during the self-study session as this might be an alternative explanation for differences in learning outcomes. Analyses showed no significant difference in the total log time spent on the digital learning environment during the self-study session (Table 5) between learners engaged in concept map completion and those engaged in concept map study (no main learning condition effect). There was also no significant difference in log time between novice and advanced learners (no main learners’ prior ability effect). Moreover, there was no interaction effect (Learning condition x Learners’ prior ability) on the time on task during the self-study phase.

**Table 5:** Mean time on task during the self-study phase (SD), and 2 × 2 ANOVA results for the effects of learners’ prior ability, learning condition and factor interaction

	Learning condition		Learners’ prior ability		2 × 2 ANOVA			
						Main effects		Interaction effect
	Concept map study	Concept map completion	Novice learners	Advanced learners	Statistical value	Learning condition	Prior ability	Prior ability x Learning condition
Time on task (min.)	95 (39)	88 (36)	92(44)	91(29)	F_(1,57)_	.524	.016	.085
					MSE	770.790	24.256	125.251
					P	.472	.898	.772
					η_p_^2^	.009	<.001	.001

MSE represents mean-square error value; η_p_^2^ represents effect size (partial eta square) calculated for all comparisons

## Discussion

In this study, novice and advanced learners studying worked examples were additionally engaged in concept map completion or concept map study to foster meaningful learning of physiotherapy intervention knowledge. The first objective of this study was to investigate which of these additional activities would be best for fostering meaningful learning of physiotherapy intervention knowledge. It was expected that concept map completion would be more effective than concept map study for fostering problem-solving skills. The second objective was to explore whether there would be differences between novice and advanced learners in benefitting from concept map completion and concept map study. It was hypothesized that both novice and advanced learners would similarly benefit from concept map completion and concept map study because physiotherapy intervention knowledge deals with ill-structured problems. The third objective was to compare the mental effort invested by learners in the guided learning phase. It was expected that concept map study would be less cognitively demanding than concept map completion. Finally, the fourth objective was to compare the mental effort invested by learners across post-tests between the concept map completion and concept map study condition. It was hypothesized that learners engaged in concept map completion would invest less mental effort while performing post-tests assessing physiotherapy intervention knowledge than learners engaged in concept map study.

### Concept map completion versus concept map study

The results of the present study suggest that concept map completion can foster more meaningful learning of physiotherapy intervention knowledge compared to concept map study in learners engaged in example-based learning with worked examples. In health profession education, meaningful learning is achieved when learners are able to use the knowledge acquired to solve clinical problems that are different from the problems studied, or presented within a context that is different from a learning context [19–21]. In the present study, physiotherapy intervention knowledge was assessed by problem-solving performance and conceptual knowledge. Problem-solving performance was evaluated by learners’ capacity to solve problems that were different from those studied. Meaningful learning was achevied in learners that were able to transfer the strategies learned to solve new problems. In this way, it could be said that the better learners were at solving new problems, the more meaningful learning they achieved.

As for the transfer of problem-solving skills, learners engaged in concept map completion outperformed those engaged in concept map study on near and far transfer performance on both post-tests (even though time on task in the guided-learning session and the self-study phase were equal), regardless of the learners’ prior ability. Concept map completion led to better near transfer than concept map study. This means that learners engaged in concept map completion were more able to directly apply the principles learned in the worked examples to solve new problems that have different superficial features but require similar strategies to those studied [23, 80]. Concept map completion also led to better far transfer than concept map study. This means that learners engaged in concept map completion were more able to solve problems that require adaptation of the problem-solving strategy learned in the examples [23, 80]. This finding shows that, when exposed to new problems, students who completed concept maps after example study were better able to recognize when the learned strategies could be used and adapt them or generate new strategies when necessary. In light of these results, educators might consider providing activities involving concept map completion to foster meaningful learning of problem-solving ability in health profession learners engaged in worked example study.

It is unclear how concept map completion can foster more meaningful learning than concept map study. In the present study, both concept map completion and concept map study allowed learners to deepen their understanding of the underlying structure of the worked examples studied. Note that students engaged in concept map completion were required to actively fill in missing information, which is something they may or may not have been able to do. As such, the completion activity may have given learners better insight into what they have and have not yet learned (i.e., a metacognitive monitoring effect; cf. Baars et al., 2013, who found that example completion increased effort and reduced students’ overestimation of their future test performance), thereby allowing them to focus on their shortcomings during subsequent example study as well as during the self-regulated learning phase [81].

It should be noted that, at first sight, these findings are at odds with those of the previous study by our team, in which concept map completion after pairs of worked examples and completion problems, did not promote transfer compared to concept map study [5]. However, in that prior study, the presence of completion problems in both conditions, may have diminished the metacognitive monitoring effect of map completion. Moreover, learners were engaged in concept map completion on several different topics and for a short time period during a single learning session. This contrasts with the present study in which learners were engaged in concept map completion on the same topic during a guided-learning session and had access to those concept maps during a self-study phase. In line with the present study, several studies have found that concept mapping was superior to traditional teaching for acquiring knowledge and fostering problem-solving abilities in health profession education [7, 20, 30]. In these studies, learners were engaged in concept mapping on the same topic in a repetitive manner over a period of time. For example, Gonzalez et al. (2008) found that medical students who constructed concept maps related to cardiovascular physiology during four sessions lasting 2 h performed better on a problem-solving exam, but not on a multiple-choice exam, than students attending the same number of sessions in a traditional course [20]. Some evidence suggests that the effectiveness of learning with concept mapping might depend on the time dedicated to this activity. In this way, nursing students engaged in creating concept maps describing clients’ physiological and psychosocial needs showed a steady increase in the quality of the concept maps generated over the course of three semesters of training [82]. Similarly, nursing students participating in concept mapping during a 16-week community-based mental health course, significantly improved their ability to identify patterns and relationships to plan and evaluate nursing care [83]. Wilgis and McConnell (2008) found an improvement in critical thinking in novice graduate nurses attending a 2-day hospital orientation in which they had to construct concept maps that reflected the main objectives of a patient’s care plan [84]. Moreover, it seems that as the number of topics addressed in concept mapping increases, so too does the amount of time that must be spent on this activity to achieve effective learning. Junior medical students engaged in a 8-week module involving concept map completion dealing with diverse common cardiovascular, renal and gastrointestinal diseases showed better understanding of the pathogenesis of diseases compared to students engaged in case-based learning on the same topics [30]. Nutrition study students who constructed, over the course of a nine-month internship, sixteen concept maps presenting standard nutrition care plans for patients with various disease states/conditions showed a significant increase in critical thinking during nutrition assessment [85]. Additionally providing concept map completion to undergraduate medical students engaged in a problem-based learning physiology course fostered meaningful learning of pathophysiological mechanisms [19]. In this study, students were engaged in six blocks (each block lasting 3 weeks, with two weekly sessions) covering the pathophysiological mechanisms of the digestive, cardiovascular, blood, respiratory, renal and endocrine systems [20]. This body of evidence suggests that additionally providing concept mapping or concept map completion can foster meaningful learning in health profession learners engaged in case-based or problem-based learning. When using this learning strategy, educators should consider the repetitive use of concept mapping or concept map completion over a certain period of time in order to achieve effective learning, particularly when various topics are addressed in a course.

### Advanced versus novice learners

Our hypothesis was that advanced and novice learners would similarly benefit from worked examples study, regardless of the learning condition (concept map study or concept map completion). This was expected because the skill to be learned — selecting optimal physiotherapy intervention while facing a clinical problem — is an ill-structured task that takes time to fully acquire. So far, only a few studies have shown an absence of the expertise reversal effect in ill-structured tasks [10, 40]. It is relevant in the planning of educational activities to investigate whether this effect is absent because it means that both novice and advanced learners would similarly benefit from learning activities combining worked example and concept mapping when the task to be learned is ill-structured (such as clinical reasoning in the health professions). Although advanced learners had some clinical experience and showed acceptable performance before participating in the learning activities, they can still learn from the examples because they have not fully acquired the task yet. Consistent with this hypothesis, far transfer performance was similarly fostered in novice and advanced learners, regardless of the learning condition. This is in accordance with previous studies that showed an absence of the *expertise reversal effect* in less structured domains [10, 40]. The expertise reversal effect is observed when learning activities benefit novice learners more than advanced learners [11]. In the present study, the expertise reversal effect was absent for far transfer performance because both advanced and novice learners showed an increased performance in this task after being engaged in the learning activities. In fact, in the present study, both advanced and novice learners had not yet learned how to solve the ill-structured problems that were used to assess far transfer performance. Consequently, both advanced and novice learners had to try to adapt the strategies they had learned, or find new strategies in order to solve far transfer problems. These results suggest that both advanced and novice learners can benefit from worked examples study for fostering far transfer performance in ill-structured domains. Given these results, educators should consider using activities based on worked examples to foster far transfer performance of ill-structured tasks in both novice and advanced health profession learners.

However, advanced learners outperformed novice learners in near transfer performance, regardless of the learning condition. This suggests that, after studying worked examples, advanced learners were more skilled than novice learners in solving problems requiring similar strategies than those studied in the examples. This can be explained by the fact that, before the learning intervention, advanced learners were already more knowledgeable and skilled in selecting electrotherapeutic currents than novice learners. In fact, advanced learners already outperformed novice learners for problem-solving performance in the pre-test phase. It is possible that advanced learners continued to maintain an avantage over the novice learners in the immediate post-test, in which near transfer performance was higher in advanced learners than in novice learners. However, this advantage seemed to vanish after the self-study phase because near transfer performance was no longer significantly higher in advanced learners than in novice learners at the delayed post-test. These results suggest that when using learning activities based on worked examples for fostering near transfer ability, educators should ensure that novice learners are given enough time to engage in these activities in order to optimally benefit from them.

As for conceptual knowledge of the physiotherapy intervention, overall results did not show a main effect of learners’ prior ability across both post-tests. This shows that both novice and advanced learners can gain conceptual knowledge when they engage in learning activities based on worked examples. However, the significant learners’ prior ability x learning condition interaction showed that advanced and novice learners did not benefit from both learning conditions in the same way. In fact, concept map study fostered more conceptual knowledge than concept map completion in advanced learners, but not in novice learners. This is in contrast to the prior study by our team in which concept map study fostered more conceptual knowledge than concept map completion in novice learners (Dyer, Hudon, et al., 2015). Why did concept map study not promote more conceptual knowledge than concept map completion in novice learners as in the prior study? This discrepancy may have been a consequence of the differences in time available for map study and map completion between the two studies. In the previous study, learners were engaged for only fifteen minutes in concept map completion or concept map study per topic (there were multiple topics), whereas in the present study, learners spent forty-five minutes completing or studying a concept map on a single topic during the guided-learning session, and could go back to that map during the self-study phase. As such, having to engage in map completion while not having sufficient time or lacking the knowledge to do so, would have played a role in giving concept map study an advantage over concept map completion in the prior study but less so in the present study.

It is remarkable, however, that map study did benefit advanced learners. From a typical expertise reversal effect perspective, one would have expected that the advanced learners, who already possessed more conceptual knowledge (see Table 1) even though the differences with the novice learners was not statistically significant, did not need the instructional guidance provided by the filled-out concept map. From this perspective, one would expect novice learners not advanced learners, to benefit from map study. However, as mentioned earlier, prior research has already shown that the typical expertise reversal effects found in highly structured tasks, may not apply to ill-structured tasks, such as reasoning about legal (Nievelstein et al., 2013) or medical (Ibiapina et al., 2014) cases, where even advanced learners still benefit from high levels of instructional guidance. The task used in the present study (i.e., selecting optimal therapeutic electrical currents in physiotherapy to treat patients with motor deficits) is also ill structured, and might take time to acquire. It is possible that because advanced learners did not fully acquire the skills for performing this task, they benefited, as did the novice learners, from the high level of instructional guidance provided by the model concept map. Moreover, one could also hypothesize that, in the present study, advanced learners would have benefited more from concept map study because the general structure of the model concept map matched well with their own cognitive schemas. In this way, the model concept map might have provided novel concepts that advanced learners were able to more meaningfully process and memorize; however, this explanation would have to be investigated in future research.

It should also be noted that there was only a slight difference in expertise between the novice and advanced learners in the present study. It is possible that the results of this study would have been different if the advanced learners had higher levels of expertise such as those found among competent or proficient healthcare professionals or experts [60, 86, 87]. The present study selected novice and advanced learners because this level of expertise is most often found in educational settings (e.g., in pregraduate students who start gaining clinical experience).. When considering healthcare professionals in their clinical practice, they are considered competent when they start to feel comfortable with professional practice. Proficient professionals understand the clinical situation as a whole and can plan long-term goals. Experts have a considerable amount of experience that allows them to have an intuitive grasp of every situation [54, 56, 87]. It is unclear how learners of higher expertise compared to advanced learners would benefit from concept map study and concept map completion. On one hand, one can hypothesize that if expert, proficient or competent learners had participated in the present study, they might have experienced the expertise reversal effect because the examples would have become redundant and imposed more cognitive load. On the other hand, more advanced learners might have benefited more from the examples if they matched well with their own cognitive schemas. It is also possible that competent, proficient and expert learners would have benefited more from concept map completion than advanced learners, because it might have been easier for them to complete the concept map if it fit their own cognitive schemas. Future research should address the role of learners’ prior conceptual knowledge and problem-solving skills in example-based learning in health profession education where problems are generally ill structured.

### Mental effort invested in the guided learning phase

In the present study, the subjective perception of the mental effort invested by learners was assessed during the guided learning phase. This measure is an estimate of the cognitive load associated with the tasks performed and is relevant when assessing learning activity efficiency in light of the cognitive load theory [53]. In fact, learning activities can be considered efficient in terms of learning processes, whether the mental effort invested is low during the learning activity [76]. In the present study, learners engaged in concept map study invested much less mental effort during the guided learning phase than those engaged in concept map completion. However, concept map study was no more efficient in terms of learning processes because it did not foster more learning than concept map completion. This suggests that the cognitive processes generated by concept map study took less effort and generated less learning than the cognitive processes induced by concept map completion. This is in line with the previous study carried out by our team showing that novice learners engaged in concept map study invest less mental effort than those engaged in concept map completion [5]. This can be explained by the fact that the cognitive actions involved in completing a concept map are more demanding than those involved in studying a concept map. Indeed, while completing the concept map used in the present study, learners must process the information in order to subsume lower-order concepts under high-order concepts and use a process of integrative reconciliation to make links between differentiated concepts [5, 6, 27, 88]. In contrast, learners engaged in concept map study might have processed the learning material in a more passive manner that did not foster in-depth learning. It seems that the cognitive processes involved in concept map completion were more effective in fostering learning because learners engaged in this activity showed better acquisition of problem-solving abilities compared to concept map study. Educators should consider that although concept map completion cognitively takes more effortl, it might foster more learning than concept map study.

### Mental effort invested in the learning outcomes tests

The subjective perception of the mental effort invested by learners was also assessed during the learning outcome tests (i.e., post-tests). There was no significant difference in the mental effort invested during the post-tests between learners engaged in concept map study and those engaged in concept map completion. This indicates that the post-tests were just as difficult cognitively in both groups. However, concept map completion led to better problem-solving performance on the post-tests, suggesting that the cognitive processes used by learners in this condition were more effective than in the concept map study condition. This is in line with our team’s prior study, in which learners engaged in concept map completion or concept map study after studying worked examples invested similar mental effort on an immediate post-test assessing conceptual knowledge and problem-solving performance [5]. Future studies should investigate how learning strategies based on concept maps should be designed to improve learning efficiency in terms of learning outcomes (i.e., higher post-test performance associated with lower mental effort invested during the test). In light of the cognitive load theory, increasing the learning efficiency of educational strategies is a desirable objective since it suggests that learners have acquired cognitive schemas that allow them to perform the task learned with much less mental effort.

#### Limitations

A potential limitation of the present study is that learners’ expertise was established based on an ability criterion (low versus high problem-solving ability) tested five months prior to the learning activities. However, analyses showed that advanced learners outperformed novice learners on problem-solving skills throughout the pre-test phase, that is, five months beforehand and just before students engaged in the learning activities. Moreover, just before participating in learning activities, advanced learners outperformed novice learners on conceptual knowledge (although not significantly). We cannot exclude the possibility that learners gained knowledge during this time. One should note that analyses suggests that novice learners might have gained more knowledge than advanced learners during the pre-test phase. As an explanation of these results, one can hypothesize that novice learners were more aware of their shortcomings after the first pre-test and were therefore more motivated to learn about the subject during the pre-test phase compared to advanced learners. However, this increased gain in knowledge among novice learners during the pre-test phase seems to have a weak influence on the study’s results since advanced learners continued to outperform novice learners on problem-solving skills (i.e., near transfer performance) on the immediate post-test performed after the guided-learning phase.

From an experimental perspective, a limitation of this study is the lack of examples in the control group only (i.e., no concept mapping). However, because this study was conducted in an educational context, it would have been unethical to withhold a study activity that could be expected to benefit learners. Another potential limitation of the present study is the evaluation of conceptual knowledge using simple multiple-choice questions. This type of question might have, at least partly, assessed factual knowledge, which is recall of facts pertaining to the domain of knowledge rather than conceptual knowledge which involves deducing the interrelations between these facts. Future studies should consider assessing conceptual knowledge with more complex multiple-choice questions, for example, questions based on short case studies that evaluate clinical reasoning or by way of essay questions. Another limitation of the present study is the fact that we did not assess what other learning activities the learners were involved in during the self-study phase. Future studies should consider providing learners with a logbook in which they note the learning activities that they participate in during the self-study phase and the time dedicated to those activities.

## Conclusion

For learners engaged in example-based learning with physiotherapy cases in a digital learning environment, additionally providing a concept map to complete, results in better performance on immediate and delayed problem-solving transfer tasks than additionally providing a concept map to study. Studying concept maps, however, seems to lead to higher conceptual knowledge (at least for advanced learners). Educators in health professions should consider providing repetitive use of concept map completion on the same topic to foster meaningful learning of problem-solving skills in learners engaged in worked examples study. Moreover, results of this study suggest that concept map completion might benefit learning of ill-structured tasks in both advanced and novice learners involved in worked examples study. Future studies should investigate how concept map completion and concept map study activities might be combined or sequenced in order to gain the benefits of both.

### Additional files

Additional file 1: Incomplete Concept Map (PDF 909 kb)

Additional file 2: Complete Concept Map (PDF 1545 kb)

## Appendix A

Prior knowledge question.

**A young professional soccer player comes to your private clinic for follow-up after suffering a muscle strain of the right quadriceps six weeks ago during a game. Your assessment reveals the presence of residual peri-muscular oedema at the quadriceps and paresis of the rectus femoris, where voluntary contraction is much weaker than the vastus lateralis and vastus medialis.**

Following your assessment, you identified the following main problems ranked in priority order:

1- Weakness (4/5) of the right knee extensors.

2- Paresis of the right rectus femoris.

3- Residual peri-muscular oedema of the right anterior thigh.

4- Decreased range of motion at the end of active extension of the right knee (quadriceps lag).

5- Decreased endurance in right knee extension.

6- Decreased function of the right knee (during sports).

7- Pain with active extension of the right knee (6/10).

**1) “What is the most appropriate electrotherapy intervention in this case?” (1 point);**

**2) “What are the optimal adjustment parameters for the electrotherapy intervention selected?” (2 points)**

## Appendix B

Worked example.

***A) Initial presentation of the clinical case.***

*Nadine, a 27-year-old female, suffered an injury 3 weeks ago while playing soccer, resulting in a grade II sprain of the lateral collateral ligament of the left knee. During this period, the sports therapist who works with her team advised her to stop playing and to follow the “POLICE” treatment method. She arrives at your physiotherapy clinic walking with one crutch under her right arm. Her left knee is still slightly swollen despite the use of ice over the last few weeks. She presents weakness in the left quadriceps during left unipodal stance and her knee occasionally collapses while walking. Nadine says that she experiences knee pain with unexpected torsion movements or when her ligament is more stretched. She would like to start an exercise program that would allow her to get back to playing soccer as quickly as possible. However, she has little confidence in her knee and still feels too weak.*

**B) Following subjective (S) and objective (O) assessments: Analysis (A).**

Here is the physiotherapist’s line of thinking while analyzing the situation.

This patient presents significant weakness of the left quadriceps due to her knee sprain. The inaccurate muscular activation of the left quadriceps is probably the cause of the collapsing phenomenon and weakness sensations reported by the patient. It also restricts unipodal weight bearing during gait. My subjective and objective assessments helped me to eliminate the unlikely assumptions of other peri-articular lesions (ligaments other than LCL, tendons, muscles) or intra-articular lesions such as meniscus tears, which could have occurred when she was injured on the field. There is no intrinsic muscle tear in the left quadriceps. Muscle testing shows moderate weakness. Reduced active range of motion is also present in the last degrees of extension. Only the LCL ligament test reproduces the pain.

My list of problems, BY PRIORITY, associated with this case is as follows: 1- Moderate weakness of the quadriceps; 2- ↓ active ROM in extension; 3- Mild oedema at the left knee; 4- ↓ function in sports activities; 5- Slight pain; 6- Patient lacks knowledge about her condition.

The wording for my physiotherapy diagnosis would be: Grade II sprain of the lateral collateral ligament of the left knee post-trauma, causing oedema and weakness of the left quadriceps, which limits walking and significantly restricts the patient’s participation in sports.

**Clinical reasoning behind the treatment plan (P):**

Here is the physiotherapist’s line of thinking for his treatment plan.

Weakness of the left quadriceps results from muscle reflex inhibition due to intra-articular oedema which developed following the LCL knee sprain. In order to allow proper weight bearing on the left limb and quickly return to sports activities, muscle contraction (within the full ROM) and proper biomechanics must be restored in this patient. To achieve this, we should train the quadriceps to achieve the last few degrees of ROM in extension with or without weight bearing using **voluntary control and strengthening EXERCISES.** These active exercises and resumption of activities involving the use of the left lower limb more than the right will also contribute to less residual oedema at the left knee.

To facilitate **SPECIFIC VOLUNTARY MOTOR RECRUITMENT OF THE QUADRICEPS** at the end of amplitude, an appropriated **ADJUVANT NEUROMUSCULAR STIMULATION METHOD** is needed. There are a few potential methods to consider. **High-voltage** stimulation is useful for precise recruitment of deep motor points, parameters of stimulation, little versatility (e.g., ramp) and unnecessary polar effects. Training in this case is too analytical and functional to benefit from high-voltage stimulation. **Russian current** (depolarized and sinusoidal wave), strong contraction, recruitment of deep or large muscles, feasibility (within your clinic context, especially if the whole muscle, including the vastus intermedius, does not contract enough or if oedema prevents optimal muscle recruitment. However, Russian current is not optimal in this case (little oedema, specific weakness at the end of amplitude) to achieve more functional stimulation during movement. (The Russian current system is bulky). Russian current is not precise enough, provides global stimulation of the quadriceps and is less flexible than conventional neuromuscular stimulation. Moreover, neuromuscular stimulation using Russian current makes it more difficult to isolate (if necessary) contraction of a specific vast of the quadriceps (medial or lateral) that could not contract properly in the last few degrees of extension (Quads lag). This young patient could also not use such current at home.

**NEUROMUSCULAR ELECTRICAL STIMULATION (NMES)** (biphasic or monophasic rectangular wave stimulation of short duration). This type of current allows for preferential recruitment of innervated fibers (similar to high-voltage current and Russian current) but the parameters are more flexible according to the targeted vast, type of contraction (concentric, eccentric), type of fibers (long or short pulse duration), ramp time (rise and fall time), contraction time and targeted training (at the end or during a portion of the amplitude). For example, NMES more easily allows for specific recruitment of isolated muscle motor points. Also, by increasing the pulse duration, we can more easily recruit the small motor fibers of the quadriceps that are difficult to contract at the end of the movement amplitude. The change in pulse duration can also have an effect on pain based on the Gate Control Theory. NMES can be used during functional activities and the patient, who understands what is expected with this treatment, could even use it at home to accelerate her recovery, after a thorough patient education session.

**Table:**

C)
**Intervention plan (P):** Conventional NeuroMuscular Electrical Stimulation (NMES).
D)
**Parameters:** Bipolar stimulation on the left quadriceps; Cathode over muscle motor points of rectus femoris (if 2 channels are available, over *vastus medialis* or *vastus lateralis* as well, if needed) and anode more proximal near quadriceps’ origin or at the femoral triangle;
Pulse duration = 300 microsec.; Frequency = 50 Hz (or more to induce tetanization); with voluntary contractions of the left quadriceps in the last few degrees of knee extension.
E)
**Key elements of the clinical case to justify the decision:**
No contraindication; Main problem: weakness; Motor control deficit; Will need a specific training program; Stimulation parameters should be programmed precisely; Can be used by patient at home (if a portable device is available).

## Appendix C

Conceptual knowledge question.

**In neuromuscular electrical stimulation, which of the following statement(s) is(are) true when it comes to pulse frequency?**

1. For a constant intensity of current (mA), if the stimulation frequency is increased, there will be an increase in tension generated by the muscle;
2. The frequency for achieving tetanization will vary from one motor unit to another;
3. Pulse frequency selection can influence muscular fatigue during prolonged muscle stimulation;
4. The sensation felt by the patient varies depending on the frequency selected.

A) 1, 2 and 3 are correct.
B) 1 and 3 are correct.
C) 2 and 4 are correct.
D) Only 4 is correct.
E) All the above statements are correct.

## Appendix D

Near-transfer question.

**You provide follow-up care at the outpatient clinic affiliated with your rehabilitation hospital. A 42-year-old male comes for a consultation visit. This patient is a construction worker and is out of work due to considerable weakness of the left upper limb after suffering an injury three months ago. He was diagnosed with partial denervation of the radial nerve and a neurosurgeon chose not to operate. The patient is referred to you for persistent weakness of the left extensor carpi without further detectable motor impairment.**

Following your assessment, you identified the following main problems ranked in priority order:

1- Partial denervation of the extensor carpi;

2- Weakness (3+/5) of the left extensor carpi;

3- Decreased left hand function;

4- Mild hypoesthesia of the subcutaneous radial nerve area of the left forearm and hand;

5- Mild intermittent pain (2/10) along the radial nerve;

6- Mild interstitial oedema of the left hand.

A- **What is the most appropriate electrotherapy intervention in this case? (1 point)**
B- **What are the optimal adjustment parameters for the electrotherapy intervention selected? (2 points)**
C- **What key characteristics of the case justify the intervention selected? (2 points)**

## Appendix E

Far-transfer question.

**A patient presented with significant interstitial edema of the right hand after the complete removal of internal fixation devices for multiple hand fractures 4 days ago. The X-ray report shows complete consolidation of the fractures and no evidence of metal in the hand. The oedema appears to have resulted in prolonged immobilization of the right upper extremity for 2 months. This patient was referred to you for rehabilitation of the right hand.**

Following your assessment, you identified the following main problems ranked in priority order:

1- Significant pitting oedema of the right hand;2- Significant decrease in active and passive mobility of all right hand movements;3- Moderate pain in the right hand on active and passive mobilization;4- General weakness of the right hand;5- Reduced function of the right hand.

A- **What is the most appropriate electrotherapy intervention in this case? (1 point)**
B- **What are the optimal adjustment parameters for the electrotherapy intervention selected? (2 points)**
C- **What key characteristics of the case justify the intervention selected? (2 points)**
