# Supplementary material for: Fostering clinical reasoning in physiotherapy: comparing the effects of concept map study and concept map completion after example study in novice and advanced learners
Source: BMC Med Educ. 2017 Dec 1;17:238. doi: 10.1186/s12909-017-1076-z (PMC5709960; doi:10.1186/s12909-017-1076-z)
Supplement: Supplementary file 1 — Incomplete Concept Map (PDF 909 kb) [file 12909_2017_1076_MOESM1_ESM.pdf]

**How do you choose an appropriate electrotherapeutic current for a motor deficit?**

**Voluntary muscle activation disorders**

Central nervous system (CNS)

Musculoskeletal system

Peripheral nervous system (PNS)

Denervation

Apraxia

Spasticity

Incoordination

Muscular system damage

Soft tissue damage (tendons/ligaments/capsule)

Partial

Total

Pathological synergy

Control disorder (agonist/antagonist)

Muscle reflex inhibition by pain

**Functional movements rehabilitation**

**Conventional neuromuscular stimulation**

**Monophasic triangular wave**

***Muscle weakness***

***Muscle injury***

***Superficial muscles or midplane***

***Small muscle***

**Inhibition of the agonist muscle (spastic muscle)**

**Russian current**

***Nerve motor point***

***Muscular motor point***

***Deep muscles***

***Large muscle***

**Monophasic rectangular wave**

**Recruitment of the antagonist muscle (of the spastic muscle)**

**High voltage**

**20 passive contractions**
**40 active contractions**
**Pulse duration: duration of the triangular impulsion at or beyond the threshold of accommodation**
**OFF: 5000 ms**

**Neurostimulation of the antagonist muscle**

**Pen electrode**

**ENDURANCE**
**Frequency: 20 Hz**
**ON/OFF 1:2**

**Strength**
**Frequency: 50 Hz**
**ON/OFF 1:5**

**Functional Electrical Stimulation**

**Bipolar (origin/insertion)**

**Monopolar (pen electrode)**
