# Supplementary figures and images for: Fostering clinical reasoning in physiotherapy: comparing the effects of concept map study and concept map completion after example study in novice and advanced learners
Source: BMC Med Educ. 2017 Dec 1;17:238. doi: 10.1186/s12909-017-1076-z (PMC5709960; doi:10.1186/s12909-017-1076-z)

# How do you choose an appropriate electrotherapeutic current for a motor deficit?

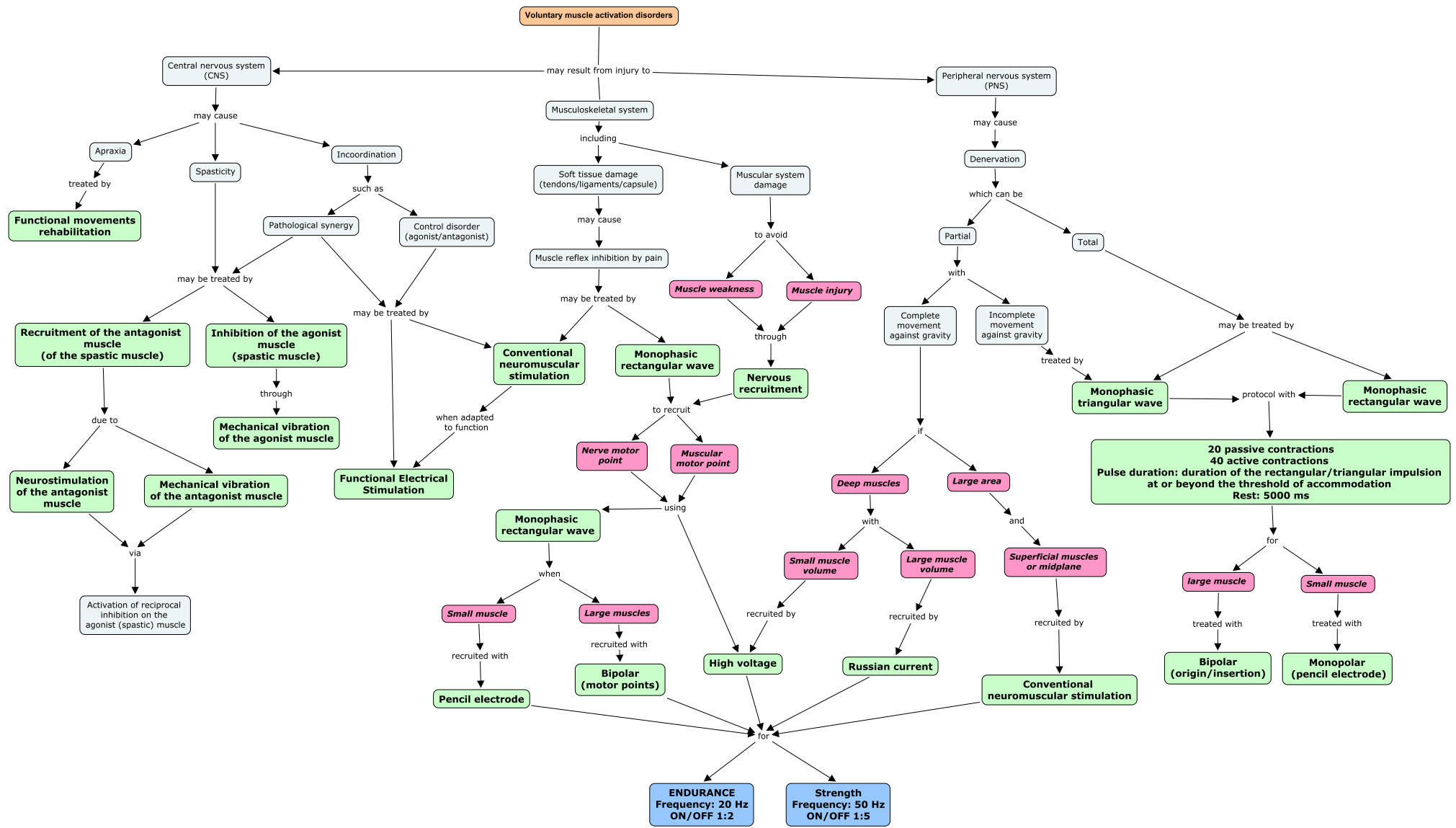

Supplement: Supplementary file 2 — Complete Concept Map (PDF 1545 kb) [file 12909_2017_1076_MOESM2_ESM.pdf]
